# Culturally and developmentally adapting group interpersonal therapy for adolescents with depression in rural Nepal

**DOI:** 10.1186/s40359-020-00452-y

**Published:** 2020-08-12

**Authors:** Kelly Rose-Clarke, Indira Pradhan, Pragya Shrestha, Prakash B.K., Jananee Magar, Nagendra P. Luitel, Delan Devakumar, Alexandra Klein Rafaeli, Kathleen Clougherty, Brandon A. Kohrt, Mark J. D. Jordans, Helen Verdeli

**Affiliations:** 1 Department of Global Health and Social Medicine, King’s College London, London, WC2B 4BG UK; 2 Transcultural Psychosocial Organization Nepal, Kathmandu, Nepal; 3 Institute for Global Health, University College London, London, UK; 4 Psychological Services, Ruth and Allen Ziegler Student Services Division, Tel Aviv University, Tel Aviv, Israel; 5 Global Mental Health Lab, Teachers College, Columbia University, New York, NY USA; 6 Department of Psychiatry and Behavioral Sciences, and Department of Global Health, George Washington University, Washington, DC, USA; 7 Centre for Global Mental Health, Institute of Psychiatry, Psychology and Neurosciences, King’s College London, London, UK

**Keywords:** Depression, Adolescent, Interpersonal therapy, Nepal, Cultural adaptation

## Abstract

**Background:**

Evidence-based interventions are needed to reduce depression among adolescents in low- and middle-income countries (LMICs). One approach could be cultural adaptation of psychological therapies developed in high-income countries. We aimed to adapt the World Health Organization’s Group Interpersonal Therapy (IPT) Manual for adolescents with depression in rural Nepal.

**Methods:**

We used a participatory, multi-stage adaptation process involving: translation and clinical review of the WHO Manual; desk reviews of adaptations of IPT in LMICs, and literature on child and adolescent mental health interventions and interpersonal problems in Nepal; a qualitative study to understand experiences of adolescent depression and preferences for a community-based psychological intervention including 25 interviews with adolescent boys and girls aged 13–18 with depression, four focus group discussions with adolescents, four with parents/caregivers and two with teachers, six interviews with community health workers and one with a representative from a local non-governmental organisation (total of 126 participants); training of IPT trainers and facilitators and practice IPT groups; and consultation with a youth mental health advisory board. We used the Ecological Validity Framework to guide the adaptation process.

**Results:**

We made adaptations to optimise treatment delivery and emphasise developmental and cultural aspects of depression. Key adaptations were: integrating therapy into secondary schools for delivery by school nurses and lay community members; adding components to promote parental engagement including a pre-group session with the adolescent and parent to mobilise parental support; using locally acceptable terms for mental illness such as *udas-chinta* (sadness and worry) and *man ko samasya* (heart-mind problem); framing the intervention as a training programme to de-stigmatise treatment; and including activities to strengthen relationships between group members. We did not adapt the therapeutic goals of IPT and conserved IPT-specific strategies and techniques, making edits only to the way these were described in the Manual.

**Conclusions:**

Group IPT can be adapted for adolescents in Nepal and delivered through the education system. A randomised controlled trial is needed to assess the impact and costs of the intervention in this setting. Future research in LMICs to adapt IPT for adolescents could use this adapted intervention as a starting point.

## Background

Depression is a major cause of morbidity among adolescents [1]. Without intervention it can lead to severe social and educational impairment in the short term, and comorbid chronic disease, further mental health problems and reduced earning potential in adulthood [2–5]. Adolescent mental health has been neglected by the global health agenda and the treatment gap is large, especially in low- and middle-income countries (LMICs) where most adolescents live [5].

The World Health Organization (WHO) recommends psychological therapies as a possible first line treatment for depression [6]. Potential therapies are cognitive behaviour therapy, interpersonal therapy (IPT) and family therapy which have been used in Europe and the US for more than a century [7]. In these countries there is substantial evidence for their effectiveness in clinical and community settings, across different age groups and populations [8–11]. More recently psychological therapies have been tested in LMICs and found to be beneficial, though there is a paucity of research among child and adolescent populations [12, 13]. Logistical challenges to delivering therapies in these settings include the lack of trained personnel to refer patients, facilitate therapy, and train and supervise therapists. These challenges have been met by training non-specialised health workers and lay people to detect depressive symptoms [14] and deliver psychological therapy [15], and by providing clinical supervision via teleconference or online supervision forums [16]. Group-based rather than individual therapies could also be more cost-effective. Moreover, the WHO published a manual for the delivery of IPT by lay facilitators in low-resource community settings using a group-based approach [17].

Aside from the logistical challenges of delivering therapies in LMICs, critics have questioned the applicability of psychological therapies developed in so-called “Western” settings, their cultural relevance, and their potential to undermine local healing practices [7]. Two meta-analyses reported that therapies that have been adapted to the local culture may be more effective than those that have not [18, 19], though a third found no evidence for an association between adaptation to the local situation and effect size [20]. The level of cultural adaptation can vary from mainly logistical or *peripheral* modifications, to deeper changes to *core* content [21]. Common areas for adaptation include language (literal translation and the use of colloquial expressions in place of technical terminology), use of stories, idioms and local symbols to convey meaning, and integration of local remedies and practices into the therapy [22]. Studies have adopted a variety of approaches to cultural adaptation, ranging from haphazard, non-empirical methods, to more systematic approaches using a conceptual framework and engaging local practitioners and community members [22–24].

There are few detailed descriptions of evidence-based cultural adaptations of psychological therapies in LMICs, and even fewer describing adaptations for adolescents in these settings [22, 25]. In this study we conducted the first cultural adaption of the WHO Group IPT Manual for adolescents with depression in Nepal. Using a multi-stage, participatory process we aimed to adapt group IPT for a rural population through a scalable and sustainable delivery platform. Here we describe the theory and methods involved in the process and detail the adaptations made.

## Methods

Research was conducted between October 2018 and November 2019 and implemented by Transcultural Psychosocial Organization (TPO) Nepal, a non-governmental organisation (tponepal.org). We obtained ethical approval from the Nepal Health Research Council and King’s College London Research Ethics Committee.

### Setting

Adolescents in Nepal are at high risk of depression due to a complex picture of recent and historical trauma (two major earthquakes in 2015 and a 10-year civil war between 1996 and 2006) on a background of socio-economic deprivation. Mental health services are limited, and mainly located in secondary and tertiary care centres in Kathmandu [26]. Specialised child and adolescent mental health services are virtually non-existent.

Our research took place in Sindhupalchowk, a mountainous district on the Nepal-Tibetan border. Most of its population (c.288,000) live in remote villages accessed by underdeveloped roads or on foot. Agriculture is the main source of income. Remittances from internal and international labour migrants are also important [27]. The main religions are Hinduism (59.0%) and Buddhism (38.0%) [28]. Reflecting the broader national picture, the population in Sindhupalchowk is hierarchical and stratified according to caste/ethnic group. The largest group is Tamang (34.2%), followed by Chhetri (18.2%), Newar (11.1%) and Brahman (10.3%) [29]. Across the district access to public health facilities is limited: there is one government hospital, three primary health care centres and 76 health posts which are mainly accessible to urban communities and those living close to a major road [30]. Net enrollment rates for primary, lower secondary and secondary level school are 95.3, 78.0, and 42.9% respectively; rates are higher among urban compared to rural populations [29]. Sindhupalchowk was one of the districts most severely affected by the 2015 earthquakes: 2071 people were reportedly killed and many lost family and friends as well as their homes, schools and livelihoods [31]. Research conducted in the district following the earthquakes found that 40% of adolescents reported symptoms of depression [32].

### Interpersonal therapy

IPT is a manualised, time-limited therapy developed in the US in the 1970s to treat depression [33]. Therapy focuses on addressing difficulties linked to four common problems that trigger depression: grief, interpersonal disputes, role transitions and social isolation. IPT has two key principles: (i) depression is a treatable medical condition and not the patient’s fault; and (ii) there is a reciprocal relationship between depression and interpersonal problems. Through therapy the patient is taught various techniques to help them analyse their interpersonal context, such as linking mood to event and event to mood and understanding how the style and content of communication can affect the outcome of a conversation. They are encouraged to use specific strategies to address their interpersonal problems. These include adjusting to life without the deceased (grief), appreciating both sides of an argument (dispute), mourning the loss of an old role (role transition), and changing habits to promote social engagement (social isolation). IPT was developed as an individual therapy for depressed adults, to be delivered by mental health specialists. Since then it has been adapted for a variety of clinical disorders including post-traumatic stress disorder, bipolar disorder and eating disorders. Adaptations for adolescents include school-based group therapy for adolescents with subclinical depressive symptoms [34], individual and group-based interpersonal psychotherapy for adolescents aged 12 to 18 (IPT-A and IPT-AG) [35, 36], and a practical self-help guide [37]. IPT has also been adapted for pre-adolescents aged 7–12 years (Family-Based IPT) [38].

We selected IPT for Nepal for several reasons. IPT has been shown to be beneficial for adolescents in other low-income country settings [39]. IPT’s focus on interpersonal triggers for depression is particularly relevant during adolescence when mental illness is often driven by interpersonal difficulties [11]. Based on previous research in Nepal, IPT is highly compatible with local conceptualisations of identity where people see themselves as part of a family and community before they see themselves as individuals [40]. Non-specialists can facilitate IPT and, given the dearth of mental health specialists in rural Nepal, task-shifting to non-specialists is essential for scalability [39]. IPT can also be delivered in a group format. In Nepal, group therapy may be a more culturally appropriate strategy as adolescents, especially girls, are likely to feel more comfortable meeting in a peer environment than with unfamiliar adult facilitators in a one-to-one setting.

The WHO Group IPT Manual outlines a simplified version of IPT for treating depression among adults [17]. Therapy is implemented in groups by supervised non-mental health specialists and comprises nine sessions instead of the usual 12–16 typical of individual and group IPT for adults and adolescents [41–43]. It involves one 90-min pre-group session between the patient and facilitator, followed by eight 90-min group sessions with 6–10 patients, organised into initial, middle and termination group phases. The Manual was developed to complement the WHO mental health Gap Action Programme (mhGAP), a package of care that aims to increase the availability of treatment for priority mental, neurological and substance use disorders, especially in LMICs [6]. The Manual uses simple language and includes practical exercises and case examples to illustrate IPT techniques and strategies. The brevity and group format of the IPT intervention, coupled with the accessibility of the Manual to non-specialists make it a suitable starting point for the cultural adaptation of IPT in a low-resource setting like Nepal.

### Cultural adaptation approach

Various theoretical frameworks for cultural adaptation exist [23, 44–47]. We selected the Ecological Validity Framework because it has been widely used to adapt psychological therapies including IPT for Puerto Rican adolescents [48], and in Nepal to guide the adaptation of Problem Management Plus (PM+) [49]. The Framework outlines eight domains for adaptation: context (the socio-economic and political environment in which therapy occurs), persons (the patient-therapist relationship), treatment goals, methods (how goals are achieved), language, concepts (how mental health problems are conceptualised and communicated), metaphors (concepts and symbols) and content (values, customs and traditions) [45, 50]. We added a ‘developmental’ domain to account for the developmental needs and preferences of adolescents. We made adaptations to the WHO Group IPT Manual under each of these nine domains.

**Fig. 1:**
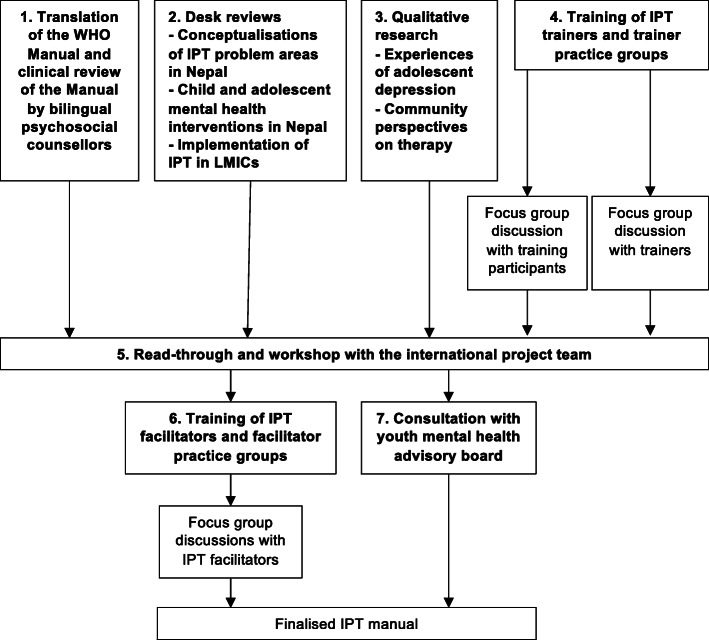
Intervention adaptation process

Our adaptation procedure is outlined in Fig. 1 and included the following steps:

1. ***Translation and clinical review of the WHO Group IPT Manual:*** A Nepali bilingual psychosocial counsellor translated the Manual from English into Nepali. Project clinical supervisors critically reviewed the translated Manual and identified potential adaptations under each adaptation domain.
2. ***Desk review:*** We conducted three desk reviews. The first aimed to review Nepali anthropological literature pertaining to the four IPT problem areas to understand how these problems are experienced and conceptualised in Nepali society. We identified and extracted data from 10 papers in total. The second sought to establish the evidence base for child and adolescent mental health interventions in Nepal in order to identify components of successful interventions which could be relevant for IPT. We identified four relevant papers. The last review explored previous IPT interventions in LMICs to understand how the therapy had been implemented, including the delivery agents, training and supervision procedures, therapy structure and measures. We identified eight studies using IPT in LMICs. In all reviews, we used key terms (e.g. ‘depression’, ‘interpersonal therapy’, ‘adolescents’) to search online databases and consulted with members of the project team who have published extensively in national and international journals on the topic of mental health in Nepal.
3. ***Qualitative research:*** We conducted a qualitative study to understand experiences of depression among adolescents in Sindhupalchowk and to explore local community perspectives on group psychological therapy. Data were transcripts from: 25 semi-structured interviews (SSIs) with adolescent boys and girls aged 13–18 with depression; four focus group discussions with adolescents aged 11–19, four with parents/caregivers, and two with teachers; six SSIs with community health workers and one with a representative from a local non-governmental organisation (total of 126 participants). Participants were identified through school teachers or local community stakeholders. Adolescents with depression were those that scored 14 or more on the Depression Self Rating Scale and four or more on a local functional impairment tool [51]. Topic guides included questions to elucidate feelings, mood and behaviour related to depression, coping strategies, potential intervention facilitators and barriers, and preferred intervention delivery platforms. SSI and focus groups were conducted in Nepali. Transcripts were transcribed in Nepali then translated into English. We used the Framework Method to manage and analyse the data [52]. We coded transcripts for deductive codes related to identifying adolescents, scheduling sessions, involvement of health workers, teachers and parents, obtaining consent, and facilitators and barriers to participation.
4. ***Training of IPT trainers and trainer practice groups:*** Master trainers and authors of the WHO Group IPT Manual HV and KC led a programme to train clinical supervisors and 12 other local psychosocial counsellors, psychologists and mental health researchers. Training was competency-based and followed the apprenticeship model co-developed by HV and KC and elaborated elsewhere [53]. It included four two-hour sessions by video conference and 4 days in person covering IPT principles, techniques and strategies, intervention structure and risk management. After this we conducted a focus group to gather feedback on the training, the content of IPT, and its appropriateness for adolescents in Nepal. Clinical supervisors conducted three practice IPT groups and nine individual cases. Master trainers provided twice weekly supervision via teleconference. We interviewed clinical supervisors to explore their experiences of training in and conducting IPT.
5. ***Read-through workshop with the project team:*** We held a workshop in Kathmandu for members of the project team during which we reviewed findings from Steps 1 to 4 and proposed changes to the Manual based on these findings.
6. ***Training of IPT facilitators and facilitator practice groups:*** Clinical supervisors trained IPT facilitators in Sindhupalchowk. In pairs, facilitators led practice groups with four to seven adolescents, supervised on a weekly basis by the clinical supervisors. We ran two focus group discussions with facilitators to explore key challenges of delivering IPT in Sindhupalchowk and recommendations for further adaptation.
7. ***Youth consultation:*** TPO Nepal regularly convenes a Nepali youth mental health advisory board to provide feedback on organisational programmes. We invited board members aged 20 and younger to a consultation about IPT. To ensure representation of younger adolescents we also invited two adolescents through TPO Nepal staff members. Two males and three females aged 13 to 20 participated in the consultation, three of whom had lived experience of depression. We sought their opinions on how to describe IPT to adolescents and the wider community, and how to address issues related to confidentiality, absenteeism, and parents’ concerns about the therapy.

We proposed further changes to the Manual based on evidence from Steps 6 and 7. Adaptations were then shared among the project team for review and incorporated into a final version of the Manual.

## Results

We made a total of 152 adaptations to the WHO Group IPT Manual. The largest number of changes were under the methods domain (*n* = 45), and the least under concepts (*n* = 7), goals (n = 7) and metaphors (n = 7). Changes were mainly adaptations to optimise treatment delivery, or to emphasise developmental and cultural aspects of depression. We did not adapt the goals of IPT beyond clarifying the aims of each phase. We conserved IPT strategies and techniques, making edits only to the way these were emphasised or explained in the Manual. Key examples of adaptations under each domain are described below and in Table 1.

**Table 1:** Key adaptations to group interpersonal therapy using the Ecological Validity Framework

Domain	Description	Examples of adaptations	Rationale (and evidence base)
Context	Increase accessibility; enhance feasibility, acceptability and compliance	Integrate group IPT into the government secondary education system.	Parents are more likely to trust an intervention if it is linked to their children’s school. There are also private rooms available in schools to hold the sessions, and supportive staff to help organise sessions and recruit adolescents. Most adolescents in the study area are in school and so it will be convenient for them to attend. Out of school adolescents said they do not have a problem attending sessions held in schools. (Qualitative study) In rural Nepal there are more schools than health posts. Locating the intervention in government schools ensures an equitable and sustainable delivery platform, and avoids potential stigma associated with visiting health services for treatment. Previous mental health interventions in Nepal have been successfully delivered through schools. (Desk review)
		Groups should be single gender but can include adolescents from different caste/ethnic groups, and younger and older, in and out of school, and married and unmarried adolescents. The preferred group size is six to 10.	Group composition: Adolescents feel embarrassed to talk about heart-mind problems in front of members of other genders but are comfortable to join groups with adolescents from different socio-economic backgrounds. (Qualitative study) Group size: A group of six to 10 adolescents is large enough for some adolescents to be absent from sessions without leaving those attending feeling exposed. It is also manageable for two facilitators. (Trainer practice groups) This number is in line with the WHO Group IPT Manual and an adaptation of group IPT for adolescents in Uganda. (Desk review)
Persons	Engaging non-mental health professionals and promoting the therapist-patient relationship	Recruit and train school nurses to facilitate IPT. Male facilitators will also be recruited from the local community.	The *One School, One Nurse* government policy seeks to appoint a nurse at every government school. The role of these nurses could be expanded to include facilitation of IPT groups. In Nepal most nurses are female however, adolescents prefer a facilitator of the same gender hence male facilitators must also be recruited from the local community. (Qualitative study)
		Facilitators work in pairs	Two facilitators are needed to manage the documents and assessments and ensure all session content is covered. (Trainer and facilitator practice groups)
Goals	Clarifying and extending goals; identifying goals relevant to adolescents in Nepal	Include aims for each phase of group sessions (Table 2)	Aims are missing from the WHO Manual and it would be helpful to clarify these to focus the sessions and support facilitators. (Read-through and workshop with the project team)
Developmental	Accounting for abrupt changes in mental state and high reactivity among adolescents; engaging parents and caregivers; ensuring content is relevant for adolescent age group	Include a second pre-group session with the adolescent and their parent/caregiver, ideally at the adolescent’s home. The session will use a strengths-based approach and take on the following structure: describe group IPT as a life skills programme, explain how will it help and state that it does not involve money, tuition or medical care; highlight the importance of confidentiality and that the adolescent will not be able to discuss problems that others bring to the group with members of their family; obtain permission for the adolescent’s participation in the group; describe how the parent or caregiver can support the adolescent.	Parents were anxious about what was happening in the groups, unaware of the potential benefits, and not supporting their children to attend. Engaging parents early in the intervention will help to mobilise their support and reassure them. (Trainer practice groups)
		*Chapter 4, Middle group phase:* Include a second meeting with parents or caregivers if the adolescent is absent for two consecutive group sessions. The aims of the meeting are to identify the barrier to adolescents attending sessions and to work together to find a solution.	Absenteeism can be an issue if the parent is not supportive of the adolescent’s participation. (Facilitator practice groups)
		*Chapter 4, Middle group phase:* Addition of the *Bhitri-Bahiri Bhawana* (meaning inside/outside feelings) technique which prompts adolescents to differentiate between the feelings they project to others and their ‘true’ inner feelings.	Adolescents had difficulty expressing their emotions during group sessions. (Facilitator practice groups)
		*Chapter 4, Middle group phase:* In the third group session each group member should develop a severe distress safety plan akin to a suicide safety plan. Facilitators should be reminded that suicide may be one of the first topics they have to discuss, possibly even in the pre-group sessions.	Due to potential abrupt changes in the mental state of adolescents, suicidality may present suddenly and adolescents should have a plan in place to help them manage such thoughts. Completing a severe distress safety plan as a group activity will help to ensure that all group members understand and are prepared. (Training of trainers)
		*Chapter 5, Strategies for disputes:* The WHO Manual describes three stages of disputes: still negotiating, being stuck or ending the relationship. Where it is desirable for a relationship to end, the individual is encouraged to end it, mourn and move on. Among adolescents we should expand the definition of ‘ending the relationship’ to include shifting the caring responsibility (e.g. from a parent to an aunt) and accepting the situation and finding coping strategies.	Ending the relationship between adolescents and their parents may not be possible or appropriate, and other solutions are required. (Read-through and workshop with the project team)
		*Chapter 5, Strategies for disputes:* Add a strategy to help adolescents manage anger. Ask the participant what they do when they are angry. Explain that anger is a natural emotion. Ask the participant if their anger had a positive or a negative effect. If negative, ask the group members for tips about how the participant can manage their anger so that it has a positive effect.. Use role-play to practice anger management. The Gestalt Empty Chair Technique can also be used. This involves participants imagining the person with whom they have a conflict and thinking about what they would say to them.	Managing anger is one of the main barriers adolescents face when trying to resolve disputes (Trainer practice groups)
Language	Ensuring translation is harmonious with Nepali language; use of local idioms of depression; replacement of technical terms with colloquialisms	Throughout the Manual, change the word *depression* to *udas-chinta*. Introduce udas-chinta as one type of *heart-mind problem*.	Although some adolescents understand the term depression, udas-chinta (meaning sadness-worry) is preferred because it: i) is Nepali, (ii) reflects the high prevalence of depression/anxiety comorbidity in this population, (iii) parents may link anxiety to the upcoming school exams and be more likely to support adolescents’ attendance. Heart-mind problem is a local, non-stigmatising term for psychosocial problems. (Qualitative study)
		*Chapter 2, Individual session:* The facilitator discusses with the adolescent how to create an environment that will “help recovery from depression”. Change to “help you to improve your depression”.	‘Recovery’ is translated as ‘healing’ in Nepali which is an unrealistic therapeutic goal. (Clinical review of the WHO Manual)
		*Chapter 3, Initial Group Phase:* The word ‘common’ is used to describe depression. Replace this word with the phrase “many people have depression - about one in four”.	Direct translation of ‘common’ in Nepali is ‘normal’. Whilst depression is common it is not considered ‘normal’. (Training of trainers)
Concepts	Using Nepali concepts of mental ill health, including somatic, social and religious concepts; addressing locally relevant stressors	*Chapter 1, Introduction:* In the explanation of the social isolation problem area, include local examples, e.g. an adolescent from the Dalit caste or a minority religion who is being excluded from friendship groups and school activities.	Social isolation is a concept that facilitators may find difficult to understand and explain. Local examples will aid understanding. (Desk review)
		*Chapter 1, Introduction:* Add content to help facilitators to understand the consequences of depression for adolescents, specifically its effects on education friendships and relationships, as well as long term health, social and economic outcomes. Link depression to healthy development and child protection.	The WHO Manual lacks information about the relevance of depression for adolescents. Linking depression to educational outcomes will be a motivating factor for parents to send their child to the groups. Communities may not be aware of the health and social benefits of improving depression. (Read-through and workshop with the project team)
		*Chapter 2, Individual session:* When inviting an adolescent to join the group, the facilitator should describe IPT as a life skills training programme	In the community adolescents are likely to experience stigma associated with accessing treatment for a mental health problem. Describing IPT as a training programme will be more acceptable to adolescents and their families and will help to promote recruitment. (Trainer practice groups)
		*Chapter 2, Individual session:* Add an instruction to the facilitator to ask the adolescent if they are experiencing any kind of physical or sexual abuse. Give examples of how to ask about this. Remind facilitators that adolescents may not feel confident talking about abuse with facilitators when they first meet and that the facilitator should be prepared to enquire again in later sessions. Include a locally relevant plan in the Manual to manage abuse and provide focussed facilitator training on this.	In Nepal, violence against children and adolescents is common. IPT is unlikely to benefit adolescents who continue to live in violent homes and require additional input from e.g. child protection services. (Facilitator practice groups)
		*Chapter 5, Strategies for dealing with life changes:* Identifying the old role and mourning its loss is a strategy for life changes. Elaborate on this metaphor of mourning a previous role. Add “What can you do that is like grieving to help you come to terms with the role change? Take the time to feel sad. It’s OK to feel sad.”	The metaphor may not be clear to participants or facilitators and requires further explanation. (Read-through and workshop with the project team)
Methods	Promoting adolescent engagement; adapting the intervention structure; adapting how depression is monitored; adapting IPT techniques and strategies	Increase the number of group sessions from eight to 12.	Two RCTs have evaluated group IPT for adolescents in LMICs involving 16 group sessions, however we expect an intervention of this duration to be unacceptable to adolescents in Sindhupalchowk and would incur high drop-out rates. Trainer practice groups suggested that adolescents take 4–5 session to get comfortable discussing openly in the groups and therefore eight sessions would be insufficient. (Trainer practice groups; Desk review)
		*Chapter 3, Initial group phase:* As a warm-up exercise, begin each group session with the *Prativa Dekhaune Kriyakalap* (meaning share a talent) activity. Each session begins with a group member sharing their talent (e.g. singing, dancing, storytelling, telling jokes, talking about an interest, hobby or person who is important to them). The facilitator shares their talent in the first session and asks for volunteers for the next session.	Adolescents said they wanted fun games to play during the group sessions. This warm-up activity will also help adolescents to get to know each other (Qualitative study)
		*Chapter 3, Initial group phase:* Add the *Mero Mon* (My Heart-Mind) game – The facilitator gives an example of a positive or negative event (e.g. mother is cooking your favourite food for dinner). Adolescents have a handout with different emoticons and point to the one that fits how they feel about the event (e.g. a happy, smiling emoticon). The facilitator then asks, how does the problem make you behave (e.g. running to reach home quickly in order to eat the food).	In practice groups adolescents were finding it difficult to link their mood to IPT problem areas. This is a fun activity designed to help with this (Trainer practice groups)
		*Chapter 3, Initial group phase:* All group members are given a *dainiki* (diary) to help them review progress, record useful information from the group discussions and any tasks to do outside sessions, and provide contact information for facilitators and other relevant services. The dainiki should be colourful and engaging. Facilitators should also provide stickers and pens for adolescents to personalise their dainikis during the sessions.	Adolescents want an incentive to attend the groups and help to remember the timing and dates of group sessions (Qualitative study). Having a booklet where adolescents can keep all of the key information will empower them and provide them with something to refer to beyond the groups. (Trainer practice groups)
		*Chapter 4, Termination group phase:* Celebrate the end of the group with the *Mitho Samjhana* (sweet, unforgettable memories) activity: each adolescent sticks a piece of paper to their back; adolescents take turns to write positive words on each other’s back.	Adolescents said they wanted fun games to play during the group sessions. (Qualitative study)
Metaphors and content	Using stories and local examples; incorporating local values, customs and practices into the Manual content	*Chapter 6, Suggestions for Facilitators:* Add additional content to help facilitators to address the following challenges/crises: argument between group members, disclosure of abuse, group member runs away from home, elopement, illness of group member or relative, and parents refusing to send adolescent to the session.	These are challenges that we expect facilitators will have to manage. (Facilitator practice groups)

*IPT* Interpersonal therapy, *WHO* World Health Organization, *LMICs* low- and middle-income countries, *RCT* randomised controlled trial

### Context: enhancing accessibility, feasibility, acceptability and compliance

We adapted the therapy for delivery through the government secondary school system for several reasons. First, in the qualitative study parents told us that they were more likely to be supportive and trusting of an intervention delivered through schools, especially if it was perceived to support their children’s education. In-school adolescents said it would be easy for them to attend sessions at school because it is where they spend most of their time, and out of school adolescents said they felt comfortable attending sessions in schools. Second, delivering the intervention through schools is potentially de-stigmatising as adolescents do not have to visit mental health services to obtain treatment. Third, the education system is a potential way to scale up IPT across Nepal and previous mental health interventions have been successfully delivered through schools in this setting [54, 55].

Through the qualitative study we obtained information about adolescents’ preferences concerning the composition of groups. They felt that groups should be single gender because they would not feel comfortable talking about their problems in front of members of other genders.

### Persons: engaging non-mental health specialists

We recruited and trained three nurses as IPT facilitators because a new national policy aims to appoint a nurse at every government school and their role could plausibly extend to the provision of student mental health care. We also recruited six community lay workers including three males since adolescents in the qualitative study said they preferred a facilitator of the same gender and the majority of nurses in Nepal are female. Facilitators worked in pairs on the basis of trainers’ and facilitators’ experiences of running practice groups and the need for two people to manage all the documents and provide support during sessions.

### Treatment goals

Based on the clinical review of the Manual (Step 1) we made adaptations to clarify the over-arching goals of IPT: (i) to decrease depressive symptoms; (ii) to improve interpersonal functioning by enhancing communication skills in significant relationships; (iii) to regulate emotions to stabilise mood and relationships; and (iv) to develop interpersonal relations with peers. We also clarified aims related to the initial, middle and termination group phases (Table 2).

**Table 2:** Aims for each phase of group interpersonal therapy for adolescents with depression in Nepal

Phase	Aims
Pre-group	Session 1: adolescent 1. Explore the IPT problem area 2. Help the adolescent to link depression and IPT problem areas 3. Gather information about key interpersonal relationships and history of depression Session 2: adolescent and parent/caregiver 1. Obtain the parent/caregiver’s consent and mobilise support for the adolescent’s participation in groups 2. Continue to strengthen rapport with the adolescent and start to build trust with the parent/caregiver
Initial	1. Help group members to feel safe and comfortable with each other so they feel able to share their experiences of depression 2. Encourage group members to review their IPT problem areas and goals 3. Explain how IPT works and create hope of recovery
Middle	1. Continue to make group members feel comfortable and encourage them to share experiences of depression 2. Help group members to listen to each other and offer ideas for dealing with problems 3. Encourage group members to try out new ideas 4. Help group members to act in a caring way towards one another 5. Continue to show that there is hope and that each group member can make changes in their life and feel better
Termination	1. Review what has happened during treatment and if/how issues related to the IPT problem area were resolved 2. Support group members to celebrate their success and say goodbye to each other 3. Make plans about how each group member can address any future or recurring problems

*IPT* interpersonal therapy

### Developmental appropriateness for adolescents

**Fig. 2:**
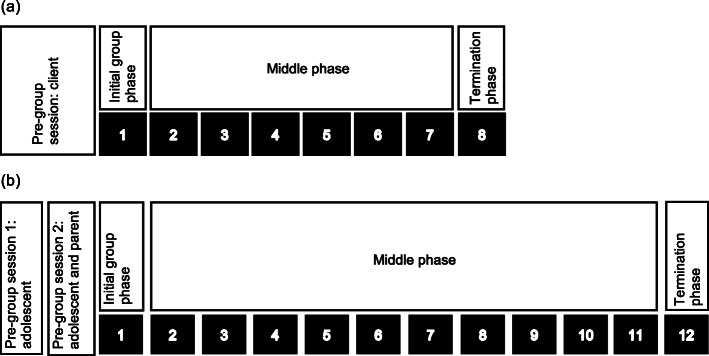
Structure of the World Health Organization group interpersonal therapy intervention (**a**) and of the adapted intervention for adolescents in Nepal (**b**)

We split the individual pre-group session into a session with the adolescent alone at school (pre-group session 1), and a session with the adolescent and their parent, ideally at the adolescent’s home (pre-group session 2). Figure 2 presents the structure of IPT in the WHO Manual (2a) compared to the adapted Nepali Manual (2b). The purpose of pre-group session 1 was to explore the IPT problem area, help the adolescent to link their depressive symptoms to the problem area, and gather information about key interpersonal relationships and the history of their depression. Pre-group session 2 involved obtaining parental consent for the adolescent’s participation in the group, mobilising support within the family, and building rapport with the adolescent and parent prior to the group sessions. The decision to split the pre-group session was based on clinical supervisors’ experience in practice groups that it was important for facilitators to gain parental support to ensure adolescents attended the group sessions.

A key IPT strategy for managing disputes is to help the patient establish the stage of the dispute: still negotiating (parties are trying to resolve the dispute), being stuck (the dispute appears to have met an impasse), or dissolution (one or both parties want the relationship to end). In practice groups with facilitators and trainers, disputes commonly involved parents. In these disputes it was rarely possible or appropriate for adolescents to end the relationship with their parent. In line with an existing adaptation of IPT for adolescents [35], we therefore expanded the definition of dissolution to include shifting caring responsibilities from the parent to another responsible adult and accepting the situation and identifying coping strategies.

Clinical supervisors running practice groups found that adolescents had difficulty expressing their emotions so we developed a technique called *Bhitri-Bahiri Bhawana* (meaning inside/outside feelings), which prompted adolescents to differentiate between the feelings they projected to others and their ‘true’ inner feelings. Since anger was the main challenge in managing IPT problems related to disputes, we encouraged adolescents to reflect on the impact of their anger and to consider how anger could be used to have a positive effect. We also included some breathing and relaxation techniques.

### Language

We replaced technical terms with colloquialisms and local idioms of depression. *Udas-chinta* (meaning sadness-worry) is a local non-stigmatising term that describes a depression-like syndrome. Findings from the qualitative study suggested that adolescents commonly experienced *chinta* (worry) due to school exams and that an intervention to reduce chinta was perceived to be beneficial for their education. We framed udas-chinta as one type of *man ko samasya* (heart-mind problem) [56]. The heart-mind is the organ of emotions, memories, and desires. Discussing mental health in terms of heart-mind problems has been found to be less stigmatising than clinical terminology (e.g., mental illness, Nepali: *maanasik rog*) and is less stigmatising than other cultural concepts of distress such as brain-mind problems (*dimaag ko samasya)*. The concept of heart-mind problems has also been used successfully in prior mental health programmes for young people in Nepal [57].

### Concepts of mental illness and locally relevant stressors

Studies identified through the desk review suggested that mental ill health is highly stigmatised in Nepal [56, 58]. We therefore sought to frame IPT as a life-skills training programme, emphasising its potential benefits for managing school pressures, future relationships, and personal development.

During practice groups, facilitators found that physical and sexual abuse against adolescents was common. We added content to the Manual to ensure facilitators pro-actively asked about abuse and included a procedure for assessment, management and referral, informed by the Nepal Ministry of Health and Population Clinical Protocol on Gender Based Violence [59].

Facilitators and adolescents struggled with the concept of social isolation, possibly because many lived in extended families where it was rare for them to be alone. We therefore added further explanation and examples of isolation, for example an adolescent from the Dalit caste group who experienced discrimination in the community and at school (long-term isolation), and an adolescent who started a new school and was struggling to make friends (recent isolation).

### Methods – how treatment goals are achieved

Our experience from practice groups suggested that adolescents took around four sessions to feel comfortable and contribute to group discussions, and that the eight group sessions outlined in the WHO Manual would be insufficient. Interventions involving 16 group sessions have been tested in other LMICs [39, 60], however the remote location of our study was a potential barrier to attendance and we anticipated high drop-out rates with 16 sessions. As a compromise we therefore increased the number of group sessions to 12 (Fig. 2).

We included games and activities in the group sessions to help engage adolescents, strengthen relationships between group members, and emphasise IPT techniques and strategies. *Mero Mon* (My Heart-Mind) is a game to help adolescents link events to feelings and feelings to behaviour. The game involved the facilitator describing a positive or negative event (e.g. mother cooking favourite food for dinner). Adolescents used emoticons to show how they felt about the event (e.g. happy smiling face) and how it made them behave (e.g. running to reach home early).

In practice groups, adolescents found it useful to have individual records of their progress, discussions, home-based tasks, and contact information. We therefore developed a *dainiki* (diary), which included a calendar of group sessions, a section to complete for each session describing what got better, what got worse and what skills have been most helpful, a template for a severe distress plan, contact details, and relevant psychoeducational information and diagrams. The facilitator brought pens and stickers to the sessions so that adolescents could decorate and personalise their dainikis.

### Metaphors and content incorporating local values, customs and practices

We replaced dialogue and case studies from the WHO Manual concerning postnatal depression, coming to terms with an HIV diagnosis, and grieving for a spouse with examples based on adolescent cases. Based on their experiences of the practice groups, facilitators identified potential crises related to elopement, bullying, illness of group members and their relatives, and arguments between group members. We therefore developed specific and culturally appropriate guidance for managing each of these situations.

## Discussion

We described the adaption of the WHO’s Group IPT Manual for adolescents with depression in Nepal. Key adaptations included: integrating therapy into the school system for delivery by school nurses and lay community members; adding a session to promote parental engagement; using locally acceptable terms for mental illness; and framing the intervention as a training programme to de-stigmatise treatment. A quantitative pilot trial is now needed to assess recruitment, participation and drop-out rates, participant trajectories, and therapist competency, in order to fully assess the feasibility and acceptability of group IPT in this setting.

A strength of our study is the participatory nature of the adaptation process which involved adolescents, parents, teachers, mental health professionals, and international IPT experts. Using a variety of methods (e.g. focus group discussions, interviews, desk reviews) we triangulated data to inform evidence-based adaptations to the Manual. We describe and justify unique and novel additions to group IPT including the dainiki and games that compliment and emphasise IPT techniques and strategies which could be relevant for other settings. Our study helps to address the lack of detailed descriptions of cultural adaptations in LMICs and is the first to adapt IPT for Nepal. The study has several limitations. The adaptations described here are based on our initial formative research. Additional adaptations to the intervention may be indicated following further piloting and a future definitive trial. We adapted IPT for adolescents in one district of Nepal. Challenges related to the remoteness of communities in this setting directly informed the adaptation though may not be relevant in other districts. We integrated culturally relevant language, customs and values into the Manual, reflecting the specific caste-ethnic composition of the population in Sindhupalchowk. These adaptations are unlikely to be generalisable across the country and the Manual may require further review prior to scale up.

We build on a previous adaptation of group IPT for adolescents in internally displaced person camps in Uganda by adapting the intervention for delivery in a school setting [39]. This is an advantage in terms of the potential for sustainability and scalability in settings with a functional education system and where adolescents face barriers in accessing health services [61]. In LMICs lacking an adequate health system schools are well positioned to offer adolescent health services because they usually outnumber health facilities and poorer communities are more likely to have schools than health facilities [62]. Moreover schools may be a less stigmatising setting for adolescents to access mental health care compared to health facilities [63]. In rural Nepal there is a concerning level of absenteeism in health facilities wherein health workers do not come to work to provide regular and predictable services. In a recent programme in Nepal to improve community referral to health facilities for mental health care, the primary care workers trained in mental health services were not present at the health facilities to provide services to persons referred [64].

Many countries are already implementing school health and nutrition programmes as a means to achieve equitable access to education, however these programmes have historically focused on nutrition, hygiene, HIV/AIDS prevention, providing safe water and improving sanitation: mental health has largely been ignored [65]. Existing research on school-based mental health interventions has mainly been in conflict-affected settings and examined prevention or promotion interventions [63]. A recent study in India reported benefits of a school-based transdiagnostic problem-solving intervention with booklets delivered by lay counsellors (college graduates) for reducing adolescents’ psychosocial problems compared to using booklets alone, though no significant reductions in internalising or externalising symptoms [66]. This problem-solving intervention is intended to be an initial brief intervention as part of a stepped care system which also includes higher intensity treatment for individuals with more severe symptoms. Group IPT could be a candidate higher intensity treatment. Approaches are also needed to reach out of school adolescents who may be more vulnerable to mental health disorders compared to their school-going peers. In Nepal, adaptations of IPT for delivery through the informal education sector or community psychosocial workers are plausible [67].

We used the Ecological Validity Framework to guide the adaptation [45]. The Framework offers a systematic way to focus attention on key aspects of a psychological therapy that are relevant to the cultural adaptation process. However, there have been criticisms of the Framework, namely that it is difficult to implement, the domains are overlapping and interpreted in different ways, and there is little evidence to determine which domains are important for promoting therapeutic outcomes [68]. Cultural adaptations of interventions should more explicitly address the proposed mechanisms of action of an intervention within the new cultural context. Enhancing social support, decreasing interpersonal stress, processing emotions and improving interpersonal skills are hypothesised mechanisms of IPT [69]. Our Nepali adaptation promotes all of these mechanisms and we worked closely with master trainers to ensure fidelity to the original IPT model.

Engagement with parents was a major theme of adaptations made under the developmental domain. The extent to which parents are involved in adolescent psychological therapy differs across settings and therapy types. In the original adaptation of IPT for adolescents parents are involved in all three phases, whereas in an adaptation to prevent depression (IPT-Adolescent Skills Training) parents are invited to attend a middle phase session [70, 71]. In a trial of group IPT for adolescents in Uganda there was no formal parental involvement, though parents may have been informally involved in helping adolescents to implement IPT strategies outside the groups and by acting as key support persons [25]. A meta-analysis reported that combined parent-child or family therapy treatments were more effective than individual child therapies, however this included younger children as well as adolescents and a diverse range of parent and child-self reported outcomes [72].

A challenge in the adaptation process was to decide on the number of sessions. Previous evaluations of group IPT for adolescents in LMICs have included 16 sessions [39, 60]. A study of individual IPT for adolescents found that a reduction in depressive symptoms of 16% or more at week 4 predicted remission at week 16 [73]. Among adults, a meta-analysis of data from studies of several psychological therapies reported that around half the patients improved within eight sessions [74]. Recent research supports the notion that around half of participants respond early to treatment (i.e. their depressive symptoms reduce by a half by the third session) and show sudden gains (absolute, relative and stable decreases in session-wise depressive symptom scores) and this is associated with positive treatment outcomes that are sustained over time [75]. There are cost-saving benefits of a shorter intervention which has implications for sustainability and scalability, especially in LMICs. A briefer intervention could also help to reduce the dropout rate. Future research should examine the optimal number of sessions for IPT in order to achieve a balance between efficacy and cost-effectiveness.

## Conclusions

We report the first adaptation of the WHO Group IPT Manual for adolescents with depression in rural Nepal. We demonstrate how a generic therapy manual developed for adults in low-resource settings can be adapted for use among adolescents within the education system, through a participatory, multistage process. Although results from the study suggest the adapted intervention is appropriate and could be beneficial in Nepal, a pilot trial followed by a randomised controlled trial is needed to fully assess feasibility and effectiveness of the intervention for uptake and scale-up in this setting.

## Data Availability

Data from this study are not suitable for sharing because of the difficulties of fully anonymising qualitative data.

## Abbreviations

IPT: Interpersonal therapy
LMICs: Low- and middle-income countries
PM +: Problem management plus
SSI: Semi-structured interview
UK: United Kingdom
WHO: World Health Organization
